# Abstracts and Gleanings

**Published:** 1888-03

**Authors:** 


### ABSTRACTS AND GLEANINGS.

**Carbuncle.**—Professor Verneuil, of Paris, thus describes his method of treating carbuncle with carbolized spray. I have used the sprays exclusively against *all* carbuncles—small, medium or large; diabetic or not; painful or painless; still closed, or opened naturally, or by artificial means. This very simple mode of treatment I found superior to all others, in stoppiug the sufferings soon and in rapidly limiting the extension of the disease.

Amongst the cases I have treated, I may cite that of a young professor of the Paris Faculty of Medicine, who died lately of diabetes complicated with albuminuria He had a very large furuncle, or boil, on his left cheek, with diffuse and deep ext ension and considerable surrounding œdema. The prognosis was grave, not only on account of the seat of trouble, but also on account of the presence of sugar, 3.5 per cent. Cardiac and pulmonary lesions rendered the administration of chloroform dangerous. I resorted to the carbolized spray. After the first application the œdema disappeared, the pain diminished and disappeared entirely in forty-eight hours; and after seven or eight days, in six of which the spray was used four times, the large furuncle was reduced to medium-sized ecthyma pustule; and it was entirely healed by the seventeenth day.

Of course, this treament will not prevent accidents which may occur when the carbuncle has given rise to an extensive sphacelus in extremely cachetic patients. But in the majority of cases, if taken early, we have in the spray an abortive treatment for carbuncle.

The manner of using the carbolized spray is known to every surgeon. A convenient apparatus is the atomizer, which is heated by alcohol, and which will work for twenty-five minutes. Such an one is sufficient for small or medium sized carbuncles, and for those which are already opened. For the large tumors, where the skin is not broken, it is better to use a more powerful apparatus, which gives a more abundant vapor and has a more considerable force of penetration. The apparatus is placed one to two feet from the skin, regulating the spray according to the sensation of the patient. I generally place nothing between the carbolized vapor and the wound, or I place there only a single thickness of transparent gauze. Up to this date I have used only the two per cent. solution of carbolic acid. I have not tried other antiseptic solutions, being contented with the results obtained with carbolic acid, which, in my experience, has never irritated the skin nor produced any symptoms of general disturbance. The number of applications of the spray is variable. Usually three or four sittings, of half an hour each, every day, are quite sufficient. Between the time of spraying, an antiseptic carbolic dressing should be applied to the lesion. The patient might find so much relief from the spraying, that the sittings could be made much more numerous—six or eight a day. The following precautions must be taken in this method:

1. Carefully protect the normal parts surrounding the carbuncle with compresses, rolled napkins, perforated cushions, or pieces of adhesive plaster perforated at the centre, according to the region which is occupied by the lesi n; at the same time protecting the patient's linen and bed clothes from becoming wet.

2. Place the patient in an easy position, so that he shall not be tired during the spraying. When the boil, or carbuncle, is at the back of the neck, or on the back, the patient should be seated on a chair, so that he can rest his folded arms on the back of the chair. When the disease is situated in the perineum, or near the anus, the lithotomy position is the best; and when it is in the lateral, lumbar or gluteal regions, the patient should lie on the side with the lower limbs flexed.

The treatment by the carbolized spray is not only very simple, but also adapted to all forms or phases of the disease, being the same from the first to the last. When used at the beginning for a small carbuncle or boil, it has a good chance of aborting it entirely. Later, when the swelling is voluminous or has a tendency to increase, it will stop its progress. Later still, when mortification and perforations of the skin have begun, it limits the sphacelus, helps to the separation of the mortified tissues, disinfects the wound, keeps it clean, and by so doing reduces the temperature and the symptoms of general disturbance. Its advantages are increased by the fact that its application does not demand the use of chloroform, and that there is no need to touch the tumor or irritate it in any way. I have said, and I repeat, that the old method of incision with the lancet was far from being innocent, that these incisions produced in enfeebled patients severe hemorrhages, which were difficult to arrest, and which necessitated the use of painful hemostatics; and that they were capable of developing septicæmia, of propagating gangrene, and of favoring the absorption of putrid matter.

Many surgeons after having opened a carbuncle freely, scrape, excise or press the spongy mass to evacuate the pus and gangrenous materials. But these proceedings are at the same time dangerous and painful, and should be absolutely avoided; for the use of carbolized spray renders them unnecessary, by disinfecting the wound.

In order to appreciate the danger of using force on a carbuncle or furuncle, one must remember that the affection is of an infectious character, and that the tumor contains pathological microbes capaple of extending on the surface, or of colonizing in the interior, by auto-inoculation, or by entering the general circulation.

This last fact is not as well known as it might be, although it is known that a carbuncle, and even a boil, is capable of giving rise to fever, general symptoms, and even visceral manifestations—albuminous nephritis, and deep abscess, for example.

In conclusion I would state the following views:

1. Furuncle and carbuncle are only different stages of one infectious disease, and are to be treated by the same therapeutical means.

2. The treatment consists in surgical interference or medical applications. The first was formerly thought to be indispensable, or at least was resorted to in the majority of cases. The second were employed as secondary measures for relief.

3. To-day surgical intervention is becoming less and less necessary, and should be reserved for exceptional cases; on the other hand, antiseptic solutions of carbolic acid, of boric acid, etc., used in a peculiar way, and especially under the form of prolonged and repeated atomization, are remarkably efficacious, while they are at the same time very simple and free from danger.

4. Sprayings, with very few exceptions, lead to a rapid recovery from the manifestations of furuncle or of a small carbuncle, and they check the disease in graver cases. They very rapidly put an end to the pain, the fever and the general symptoms; they disinfect the purulent and gangrenous spots, and assist the cleaning of the lesion and the formation of granulation tissue.

5. Sprayings may be used in any region of the body for all forms, and in all the stages of the disease. They are never dangerous, and will alone bring on a cure in the mojority of cases. They would also help greatly to the success of surgical interference, if such should be deemed necessary.

6. Finally, they prevented internal auto-inoculations and the phenomena of general infection.—*Medical and Surgical Reporter.*

**Hydrastis Canadensis in Uterine Hemorrhage.**—Dr. Hach, of Riga (*Proceedings of the Riga Society of Med. Practitioners*), has administered hydrastis Canadensis, in the form of an American fluid extract, and of a Riga tincture (the latter in doses of one teaspoonful twice a day), in 97 cases of uterine hemorrhage, and obtained either complete or partial success in 47 of them. He gave the drug in twenty-two cases of uterine fibroid, with brilliant results in 8 cases, and with partial success in the remaining 14; in 4 cases of parametritis, with good results in 3; in 10 of perimetritis, with partial improvement in 4; in 12 of metritis, with success in 7; in 14 of metritis and endometritis, with partial relief in 4; in 19 of metritis and parametritis, with success in 8; in 3 of cancer of the vaginal portion, in all of which bleeding was restrained; in 11 of congestion dysmenorrhœa, with some success in only one; and in 2 cases of climacteric metorrhagia, in both with partial success. The author found that the American preparation acts much better than the Riga one; at least, of 14 cases treated by the former, the bleeding had ceased in 9, that is to say, in all about which information could be obtained, the remaining 5 patients having been lost sight of. On the whole, Dr. Hach recommends the drug as a good means of controlling and preventing flooding of any kind. Except some cardiac palpitation in a pregnant woman after taking 30 drops of the drug, he never saw any untoward secondary effects; on the contrary, hydrastis canadensis improved the appetite and the intestinal function. Dr. von Stryk, of Riga, however, lately saw a woman in the fourth month of pregnancy, in whom abortion took place on the third day of the hydrastis treatment, the tincture has been used in daily doses of 100 drops for relief of severe cervical catarrh (Chiari's). Among other Riga practitioners, Dr. Huebner obtained satisfactory results from the drug in 3 cases of fibromyoma, while Drs. Krannhals and Hampeln, having tried hydrastis in hæmoptysis of phthisis and nephrorrhagia respectively, did not observe that it did any good.—*British Medical Journal.*

**Antipyrin as a Hæmostatic.**—At a meeting of the Societe de Biologie, of Paris, January 7, 1888, M. A Henocque calls attention to the fact that December, 1884, he described a hæmostatic influence of antipyrin which he has since demonstrated more completely, and which has been confirmed by other and independent observers. His own experiments indicate that antipyrin, in powder or in solution, produces constriction of the blood-vessels when applied to them directly. This view is opposed to that of Caravias and Gley, who believed that antipyrin dilates the bloodvessels; but it seems to be proved by the experience of Henocque.

This fact is so important that it appears worth while to describe the way in which Henocque uses antipyrin as a hæmostatic. He employs it in powder, in solution, or incorporated in an ointment or pomade. Used in powder it is applied directly to a wound, and is covered with a dry dressing of cotton or charpie. For epistaxis the powder can be snuffed up the nose; for hemorrhage from the uterus it can be applied to the cervix or to the cavity of the uterus upon a suitable tampon. For wounds he generally uses a wash containing five per cent. of antipyrin; but for deep cavities, like the nose, he thinks a twenty per cent. solution should be used. In practice he finds it convenient to use sterilized cotton or filter paper, soaked in a strong solution of antipyrin and then dried, which can be applied to a wounded surface either dry or after soaking in boiled water.

In a case of cancerous ulceration of the breast now under his care, he has used with the best results a pomade made of one part of antipyrin with three parts of vaseline (cosmoline would serve as well) rubbed into cotton cut into small segments, to make it more coherent. This he applies to the surface; when it is to be removed he loosens it with a wash containing one per cent. of antipyrin, so as to prevent hemorrhage. He changes the dressing only twice a week, and finds that it has, in this case, put an end to suppuration and to the characteristic odor of cancer.

When it is borne in mind that antipyrin is antiseptic, it will be seen that the fact that it has hæmostatic properties also, makes it a valuable addition to the armamentarium of the surgeon and of the general practitioner.—*Medical and Surgical Reporter.*

**How to Administer Chloroform.**—The best method of internally administering chloroform, according to the *Northwestern Pharmacist*, is with twice or three times its own volume of olive oil, and finally emulsifying with powdered acacia. Its pungency is thus disguised.—*Medical Digest.*

**Bacteriology and Practical Medicine.**—The following interesting survey of the position of bacteriology with respect to medicine is transcribed from the *Centralbl. fur Bacteriologie* (No. 18). It appears as an abstract, by Dr. Bujwid, of Warsaw, of papers by Dr. Hoyer, in the Polish journal *Gazeta Lekarska.* The author, who was the first to commence working at bacteriology in Warsaw, discusses the changes which medicine has undergone by the study of the parasitic origin of infectious diseases, and arrives at the following results:

All researches hitherto undertaken have aimed at learning the excitants of disease; very many of them have been discovered, and many have been profoundly studied, so that the cause of nearly all infectious diseases has been known; but bacteriology has hitherto confined itself to these limits. Practical medicine in the more limited sense—prophylaxis excepted—has gained very little therefrom, but it may be hoped that the medicine of the future will play quite a different part in consequence of the deeper knowledge of the various bacteria and their properties. Many purely empirical drugs will be rejected, and in their stead will be employed those which bear directly upon the morbific agent, or which act by strengthening the resistance of the organism. Unfortunately many questions still remain open. We know, for instance, very little of the way in which bacteria influence the physiological life of the organism. We cannot as yet determine why many micro-organisms which are introduced into the body in enormous quantities with water, air, or food, do not give rise to derangements, or in what manner the really harmful organisms disappear from the blood or organs of some animals. Of great importance for the practitioner are the facts that similar groups of disease can be excited by wholly different micro-organisms. Abscesses are produced, for instance, by the action of staphylococcus aureus and albus, streptococcus pyrogenes, micrococcus tetragenus, and others. Erysipelas following wounds depends not only on the streptococcus erysipelatis of Fehleisen, but also on other treptococci and micro-organisms. Pneumonia is not only excited by Friedlander's pneumonococcus, but a'so by other bacteria. Two very similar diseases—cholera asiatica and cholera nostras—arise from two very different kinds of bacteria. There are other facts of still greater importance, such as mixed infections. Rosenbach found many very different bacteria in the same abscess. The same is the case with septic infection of wounds. Similarly, as Wiegandt has observed, a kind of streptococcus is occasionally associated with tubercle bacilli. Dr. Dunin has shown that certain complications of typhus depend on the presence of other bacteria, etc. When all these questions are solved, then our system of diseases will also be changed; we shall then no longer group them according to symptoms, but causes. There still remain many such questions unsolved. We do not know, for example, upon what depend the different results of experiments on animals when we inject small or large quantities of bacteria. Lastly, we also know very little of the reason why individuality plays so large a part in the manifestation of disease. Very interesting but unexplained is a research pursued by Wysskowitsch. He found that bacteria which had no effect on healthy animals excited disease in other animals whose organism was slightly deranged. Thus, injections of staphylococcus excited endocarditis in animals whose heart valves were injured. When all the foregoing and many like problems are solved, then it will become more easy to employ bacteriology in practical medicine, and then we shall learn to estimate rightly the great value of this new study.—*London Lancet.*

**Intubation or Tracheotomy.**—(Max J. Stern, M. D., *International Medcal Congress*) Dr. Stern's paper is published in full in the *Medical Register* for Novvember 12, 18`7, and is based upon 953 operations by 75 operators, and 252 recoveries after intubation. He closes his paper as follows:

The grave question now remains when shall tubage be done and when tracheotomy performed?

1. All things being equal, I would always intubate where the patient is under three and a half yeals of age.

2. Between the ages of three and a half and five years, I would be regulated, of course, by individual circumstances, with a preference for tracheotomy.

3. Over five years of age I should perform tracheotomy.

4. In adults I would never tracheotomize, but willingly test intubation.

5. Among poor people, irrespective of age, I would always intubate. While it may sound harsh to draw such class distinctions, good reasons are forthcoming. The general results of intubation are about equal to those of tracheotomy. Skilled attendance, such as is always required after tracheotomy, can only be procured for considerable purchasing power, and is in consequence only available where people have means. While the operator himself may be willing to give his own valuable time, he may owe to other patients attendance that may be of as much value to them as to the child operated upon.

6. Intubation should never be performed at any age, when there is a strong probability that the trachea is crowded with membrane.

7. Where skilled assistants cannot be obtained, intubation should always be practiced.

I should not like to close the paper without a slight tribute to labors of Dr. O'Dwyer, who, through his perseverance and patient period of experimentation, has placed in our hands a procedure that will probably save many lives. Had he, upon the incipiency of the experiments, followed Bouchut's plan of immediately publishing his results, would it not again have shared a like fate? Which of us may not yet have cause for a personal debt of gratitude to this modest inventor.—*Epitome.*

**Antipyrin as a Uterine Sedative.**—M. H. Chouppe has already called attention to the good effects of antipyrin in uterine pains after parturition or in dysmenorrhœa. In proof of this he relates the following case: A woman, aged 35, was suffering from a large myoma situated in the posterior wall of the uterus, accompanied by copious hemorrhage, which reappeared after menstruation. Ergot checked the hemorrhage, but caused such severe uterine pains that it had to be discontinued. Large doses of morphine were administered; these caused the pains to disappear. but at the same time the uterine contraction was relaxed and hemorrhage reappeared. Ergot was again administered, with similar results. The attacks of uterine pains lasted two or three hours. M. Chouppe then had recourse to antipyrin. An injection, containing 2 grammes of antipyrin, was administered. At the end of twenty minutes the pains disappeared. M. Chouppe then tried the following experiment: An injection of antipyrin was given half an hour before the dose of ergot. The patient experienced no pain, although there was active uterine contraction; hemorrhage was arrested. M. Chouppe concludes that antipyrin relieves the pain caused by the uterine contraction which is produced by ergot without diminishing the contraction. He believes that it acts upon the spinal cord, and might be administered with advantage during parturition to women of an irritable temperament. —*British Medical Journal.*

**Relative Value of Antipyrin and Antifebrin.**—Dr. G. W. Barr of Bridgeport, Ill., has made a most careful clinical study of antipyrin and antifebrin on himself whilst suffering from fever complicated with malaria. He thus sums up his experience:

| ANTIPYRIN. | ANTIFEBRIN. |
|---|---|
| Lowers temperature in half an hour. | In an hour or more. |
| Effect lasts two hours. | Effect lasts six hours. |
| More diaphoretic. | More diuretic. |
| Depressing after effects. | No after effects. |
| Cerebral sedative. | Cerebral vaso-motor and muscular (?) stimulant. |
| Dose, 15 to 30 grains. | Dose, 5 to 15 grains. |
| Tolerance from continued use. | Tolerance from continued use. |

This table, he says, will suggest the selective use of the two drugs. From the patient's point of view (which is really coincident with the physician's), antifebrin is much to be preferred in continued fevers, because the dose is one small capsule instead of three; the effect lasting so long requires one-third the number of doses; the tonic stimulation excels the depression and after malaise; and the cost is one-fourth that of antipyrin. The antipyretic action of antifebrin is as strong or stronger than that of antipyrin, and its only objection is its slowness of action. In isolation and other cases where a quickly acting antipyretic is necessary, and when it has a specific action on the pathology of a disease, as is claimed in rheumatism, antipyrin, is to be preferred. Whenever one can wait an hour for the antipyretic action to begin, he greatly prefers antifebrin, and so he believes will the patient also. He regards its stimulant or tonic effect as very valuable in weak patients.—*Therapeutic Gazette.*

**Some Remedies in Diphtheria.**—Dr. Herbert L. Snow says that it is a matter of regret to him that the old-fashioned treatment of diphtheria by tincture of iron and chlorate of potassium has not become obsolete. He claims that this mixture has no effect whatever upon the diphtheritic membrane, and assists the natural course of the disease little if any.

A rapid disappearance of the exudation with general improvement of the patient is brought about by the administration of sulphurous acid in dram doses every half-hour. The vapor of the acid produces a choaky feeling, which can be largely obviated by giving it in large quantities of syrup.—*Medical Review.*

**Sulphate of Sparteine.**—The *Deutsche med. Wochenschrift*, in a review of sparteine, remarks that it is the alkaloid of broom, and belongs to the alkaloids which have no oxygen in their composition. The sulphate is employed for medical purposes, because it is more easily soluble and less easily decomposed than the other salts. It forms large, translucent, colorless rhomboid crystals, readily soluble in water, and of a bitter taste. Germain See found that it possessed a stimulating action upon the contractions of the heart, slowing its action; and that it increased the arterial tension, and also increased the flow of urine. The rhythm of the heart's contractions was restored in only a few cases. The action of sparteine was rapid, but was not maintained sufficiently long at its height to remove the disturbances in compensation; and even when given in repeated doses does not achieve any such persistent action as is obtained through the use of digitalis. Voight, however, recommends it in valvular disease, with and without the disturbances in compensation which are proper to it. He also recommends it as a regulating and ca'mative agent, in insufficiency of the heart muscle without disease of the valves, in pericarditis, and finally as an adjuvant in combination with digitalis. The dose is about $\frac{1}{3}$ of a grain, three times a day. The following formulæ are given:

℞. Sparteini sulph.............................gr. iij,
Aquæ destillat.............................f ℥ ij$\frac{3}{4}$.

M. Sig.—Twenty drops in sweetened water or wine, two to for times daily.

℞. Sparteini sulph.............................gr. iij,
Syr. aurantii cort.............................℥ iij,

M. Sig.—Dose, a small spoonful in water, four times a day.—*Medical Reporter.*

**Diphtheria of the Ear.**—Dr. Robert Barclay, St. Louis, says: Drs. A. Jacobi, A. Politzer, C. H. Burnett, and others too numerous to mention, emphasize the frequent invasion of the middle ear by naso-pharyngeal diphtheria.

References might be multiplied if the occasion permitted, or necessity demanded, but these will suffice to place you upon your guard while attending cases of diphtheria.

Let me sum up by reminding you that aural invasion by diphtheria is very insidious, usually comparatively painless, showing a marked tendency uniformly to assume the chronic form, and apt to produce very rapid and widespread destruction, with necrosis, and burrowing to neighboring parts. Among its probable effects may be expected one of the following: fetid discharges, often persistent, always annoying and disgusting. deafness, erosion of the Eustachian tube, mastoid perforation, necrosis, partial or facial paralysis (Bell's Palsy), meningitis, thrombosis, embolism, phlebitis, pyemia, cerebral abscess, and death. These dangers, in view of the insidious development of aural diphtheria, should lead you to examine the ear early and frequently in every case of diphtheria which comes under your supervision. Should you find an inflamed and bulging drumhead, even if the patient make no complaint of aural discomfort or pain, it is your duty to make an incision of the drumhead—not a "pinhole," but an opening—sufficient to permit the escape of the products of inflammation, removing them by artificial means if necessary, to check the burrowing to neighboring regions. Let the practice of syringing the fauces and nares—which you have heard vigorously recommended for cases of diphtheria with prominent naso-pharyngeal manifestations—be prudent and careful, lest you force materies morbi within the Eustachian tube, or into the middle ear. Whatever or wherever the local phenomena, pay close attention to the constitutional condition; treat your *patient;* and modifying and adapting local measures for management of diphtheria, let free drainage, cleanliness, and antisepsis be your aim.—*Epitome.*

**Medical Aphorisms.**—A correspondent of the *Canada Lancet* hits the nail on the head in the following remarks:

1. Life is short, patients fastidious, and the brethren deceptive.
2. Practice is a field of which tact is the manure.
3. Patients are comparable to flannel—neither can be quilted without danger.
4. The physician who absents himself runs the same risk as the lover who leaves his mistress—he is pretty sure to find himself supplanted.
5. Would you rid yourself of a tiresome patient, present your bill.
6. The patient who pays his attendant is but exacting; he who does not is a despot.
7. The physician who depends on the gratitude of his patient for his fees is like the traveler who waited on the bank of a river until it finished flowing, so that he might cross to the other side.
8. Modesty, simplicity, truthfulness! cleansing virtues, everywhere but at the bedside; there simplicity is construed as *hesitation*, modesty as *want of confidence*, truth as *impoliteness.*
9. To keep within the limits of dignified assurance without falling into the ridiculous vauntings of the boaster, constitutes the supreme talent of the physician.
10. Remember always to appear to be doing something—above all, when you are doing nothing.
11. With equal and even inferior talent, the cleanly and genteely-dressed physician has a great advantage over the dirty or untidy one.—*Boston Medical and Surgical Journal.*

**Salicylate of Sodium in Whooping-Cough.**—Dr. Aumiatre says (in the *Gazette Medicale de Nantes*) that he had excellent success with salicylate of sodium in whooping-cough. The dose is from two to three grains twice or thrice daily.—*Ex.*

**Laparotomy in Peritonitis.**—Dr. Johnston (in *Med. Char. Tr.*) says: Treves mentions a case reported by Buchanan, of Glasgow, (*Lancet*, Lond., 1871, i, 776), in which there was supposed intestinal obstruction. Abdomen opened; no obstruction; extensive peritonitis; abdominal cavity sponged out; recovery. Author maintains that a peritoneum which has once been inflamed will bear operative interference with greater indifference than will a membrane upon which no such morbid process has encroached. He recommends abdominal section in the treatment of acute general peritonitis, to be followed by irrigation of the serous cavity and drainage. The operation should be performed immediately after the diagnosis is established. Temporizing, he says, is futile, reckless and purposeless. The records of medicine may be searched into at great length before an instance of recovery from acute perforative peritonitis can be found. He believes, however, that there are cases of acute inflammation of the peritoneum to which this mode of treatment, or any other of like purport, would not be applicable; such as peritonitis in connection with carcinoma, tuberculosis of the serous membrane, peritonitis the outcome of general septicæmia, and extensive rupture of certain of the viscera.—*Practice.*

**Treatment of Warts by Internal Administration of Arsenic.**—Mr. B. G. Pullin, in a communication to the *Bristol Medico-Chirurgical Journal*, says that during the last two years many cases of warts on the hands of children have come under his notice for treatment. He reports three cases, which, if they are to be credited, certainly show remarkable efficacy on the part of arsenic. The first case was that of a young woman of seventeen, who had innumerable warts on her hands, which had grown with great rapidity. Some of the largest of these had attained their growth within a week or ten days; the whole skin of the hands seemed to be filled with small growths, some not visible, but easily distinguishable to the touch. Nitric acid was applied to about one-half dozen of the largest, and she was given minims of liquor arsenicalis, and returned in a week without the vestige of a wart. In the second case, that of a boy eight years old, no local treatment was used, and all the warts but one had disappeared in two weeks, under minims of the liquor arsenicalis. The remaining one was removed with the finger. The third case is similar to the second.—*Med. and Surg. Reporter.*

**Artificial Ripening of Cataracts.**—Dr. Bœrne Bettman, of Chicago, in the *Journal of the American Medical Association* (Dec. 3), says that instead of merely rubbing the lens through the cornea, after iridectomy has been performed, as recommended by Foster for the ripening of immature cataract, that he introduces a spatula through the corneal incision, if necessary behind the iris, and triturates the lens directly by gentle stroking and pressure in any direction. Of course this proceeding requires great caution. We must not by the exertion of excessive force dislocate the lens, nor by turning upon it the sharp edge of the spatula lacerate the anterior capsule. Dr. B. asserts that the distribution of opaque lens fibres and breaking up of the cortex can be seen with the naked eye during the operation, and that the lens becomes totally opaque within a few days. He gives the history of three cases in which slowly ripening cataracts were thus matured, one in forty days and the other two in three weeks.—*N. O. Med. Journal.*

**Ephedrine, a new Mydriatic.**—Prof. Nagai has extracted from the *Ephedra vulgaris* Rich., by a process whose details he promises to publish subsequently, an alkaloid to which he has given the name ephedrine. In a preliminary note on the action of the drug M. Kinnossuke Miura ascribes to a ten per cent. solution of the chlorhydrate the following advantageous mydriatic properties: The pupils are dilated in from 30 to 60 minutes after an instillation of 1 or 2 drops; the dilatation, though not complete, and though the pupil reacts slightly and temporarily, is sufficient for thorough examination; children and old people are more sensitive than adults; the accommodation is scarcely, if at all, paralyzed; the dilatation lasts from five to twenty hours from the time of instillation; no bad effects, constitutional or local, are observed after long continued use. The mydriatic effect upon the inflamed iris is very slight.—*Therapeutic Gazette.*

**The Finishing Touch.**—It is sometimes alluded to as pitiful that it is not permitted to the physician to dispose of the future of his incurable patients. There are many instances in a general practice when the kindest thing would be to put a period to the miserable existence which has ceased to be of pleasure or profit to any one. Most of us know of some such unfortunates who would welcome death as a kindly boon, and to whom the prolongation of life is an agony and a weariness. But there are moral responsibilities which forbid the physician wilfully to become an executioner. His office is to annul pain, or to terminate it, if possible, but never at the deliberate sacrifice of life. As to what would be the most just and generous conduct under these circumstances, it is not permitted us to decide practically. But, beyond a doubt, the duty is always clear to make the painful probation of the incurable as much of an oblivion as it is possible.—*Medical Register.*

**Poisoned by Flannel Underwear.**—Prof. Bohanon, of the Ohio State University, has been suffering from the effects of poison introduced into his system by wearing red flannel underclothing. A couple of weeks since the professor purchased a number of suits, and soon after putting them on commenced suffering from a burning sensation of the skin. Large blotches appeared all over his person. A physician pronounced the case one of poisoning from the red flannel suits. One of the garments was soaked in water, which was examined by the college chemist, who pronounced the solution thus obtained extremely poisonous, only a small quantity being necessary to kill a dog. The garments purchased were of the best quality, and high priced.—*Drug. Cir.*

**Salol in Typhoid Fever.**—Dr. James Barnsfather, of Dayton, Ky., (*N. Y. Medical Journal*) says that in all the cases of typhoid fever I attended this fall, in addition to the usual treatment, I prescribed the new synthetical compound, salol (salicylate of phenol), from the very commencement, and continued it to convalescence, in doses of 3 grains every three hours, with the happiest resu'ts so far. It seems to have a powerful action upon the leucomaines formed during the progress of the disease, evidently destroying to a certain extent their toxic action on the nervous centers (if I am to judge by results). I may also state that in none of my patients was the stomach disturbed by the small doses given.—*Epitome.*

**Another Cure for Consumption.**—Prof. Lutton, of Rheimes, asserts that a cure of tuberculosis can always be effected by the employment of the phosphate of copper, which, however, must be in the nascent state and soluble in an alkaline body. His formula is as follows:

℞. Acetate of copper neutral..............grammes 0.15,
Crystallized phosphate of sodium......grammes 0.75.
Glycerine and powdered licorice, of each, q. s.

This for one pill.

**The** following is an extract from a petition, written in "English as she is spoke," by a native East Indian to the governor of his Province: "That your lordship's honor's servant was too much poorly during the last rains, and was resuscitated by which medicines which made magnificent excavations in the coffers of your honorable servant, whose means are circumcised by his large family, consisting of five female women and three masculine, the last of which are still taking milk from mother's chest, and are damnably noiseful through pulmonary catastrophe in their interior abdomen."—*Medical Age.*

**Law of Proportion of Sexes.**—Kisch says that from the statistics of 556 marriages and 1972 births, he deduces the conclusion that when the husband is more than ten years older than the wife, a large proportion of males will be the result. This is especially marked when the woman is between twenty and twenty-five years old. If the woman be under twenty years old the contrary happens, and the girls are in the majority. The latter is also the case when the wife is older than the busband, or when the ages are very nearly the same. If the husband is very much older than the wife, however, the males will predominate by two to one.—*St. Louis Medical Journal.*

**Theine in Rheumatism.**—A case of chronic rheumatic pain of left shoulder joint and whole arm. Pain very severe. No relief rendered previously by salicylates, ammonium chloride, iron, quinine, or potassium iodide. Half a grain of theine above shoulder joint made him comfortable at once.—*Dr. May in The Polyclinic, January, 1888.*

**Prophylaxis of Croup.**—This subject has been studied by Dr. Dumas for years ; he has tried a great many substances with this object in view. The agent which he thinks best of is iodine given internally in quantities not exceeding 8 *drops* daily. He gives it in orange flower water, sweetened with syrup, and adds a little iodide of potassium. He quotes a number of cases where there was every reason to suspect an attack of croup, where the use of this remedy appeared to prevent the onset.—*Arch. of Gynæcology.*

**Uterine Hemorrhage.**—In a discussion upon this subject Dr. Kenyon believed that many cases of post-partum hemorrhage were due to the penicious custom of keeping traction upon the cord, the danger of which was manifest before the uterine contractions ensued. The physician should wait (Dr. Stallard) till the uterus has recovered its vigor. Dr. Anderson gives vinegar by mouth (dose not given) and finds it a very efficient agent in promoting contraction. Several physicians believed "that in post-partum hemorrage nothing was so efficient as hot water."—*Medical Sun.*

**Detention of Metallic Bodies with the Magnet.**—In the *Munchener med. Woschenschrift*, 1877, No. 15, Graser describes the case of a man from whose arm four pieces of a needle had been previously removed, and in which the presence of another piece was suspected. By the use of a strong electro-magnet the location of this piece was discovered, and it was afterward successfully removed through an incision at the spot indicated by the magnet.—*Centralblatt fur Chirurgie*, October 8, 1887.

**Chloral Hydrate in Rabies.**—Brown Sequard reports a series of experiments on rabbits and birds, in which he produced a kind of rabies by injecting oil of tansey. This rabies he was able to control by the vapor and subcutaneous injections of chloral. Brown-Sequard thinks that, from analogy, chloral is a preventive of true rabies. He refers to his previous writings on this subject, and to cases in man in which he exemplified his theory.—*L' Union Medicale.*

**Removing Particles from the Eye.**—Among the almost numberless methods of removing particles from the eye, the following is recommended as an efficient means. Make a loop by doubling a horse-hair; raise the lid of the eye in which is the foreign particle, slip the loop over it, and, placing the lid in contact with the eyeball, withdraw the loop, and the particle will be drawn out with it.—*Scientific American.*

**Canadol, a New Local Anæsthetic.**—Canadol is a hydrocarbon derived from petroleum. It is a light, volatile, exceedingly inflammable liquid, with an odor resembling that of benzine. It is not soluble in either water or alcohol. By its rapid evaporation, will lower temperature more rapidly than ether. Canadol may be used in slight operations in the same manner as the ether spray. —*L' Oculistique.*

**Some Suggestions on the Pathogenesis of Yellow Fever.**—Dr. Ignacio Alvarado, of Mexico, in a paper with this title, took the view, based largely on his experience with yellow fever at Vera Cruz, that the disease was caused by a specific microbe, and that the symptoms were due either to acid phosphate of sodium or phospho-glyceric acid set free from lecythin by the reactions produced in the blood by the microbe.

**Glycerine in Enemata.**—Fifty drops of glycerine injected into the rectum is a very efficient remedy for producing energetic and copious dejections. The action is dependent upon the property of the glycerine attracting water. There is a transfusion of water from the intestinal walls into the canal, followed by an afflux of blood to the parts and consequent desire to defecate.—*Medical Review.*

**Salicylate of Sodium in Facial Neuralgia.**—Dercum reports that in a case of facial neuralgia, rebellious to all usual treatment, he had had immediate amelioration and speedy cure from salicylate of sodium in thirty grain doses repeated every four hours. We have had good results from the drug in similar doses in hemicrania.—*St. Louis Medical and Surgical Journal.*

**A Remedy for Neuralgia.**—It is claimed that a few drops of the fol.owing—eau de Cologne, ether, chloroform aa ʒ ij—poured on a handkerchief previously wetted with cold water, and placed on the seat of neuralgic pain gives instantaneous relief. It is also very efficacious for nervous headache. A burning sensation is ſelt at first, but quickly disappears.—*Medical Review.*

**Arnica.**—The *St. Louis Medical Journal* says of arnica in such common use: As Hardy has pertinently remarked, it was never known to have done any good and it has often produced a great deal of harm. Of late years the public has, to a great extent, discarded arnica and substituted hammamelis therefor and this is an improvement in the right direction.

**Cocaine Hydrychlorate** is rapidly increasing in favor as an anæsthetic ; a great deal of minor snrgery is done without any suffering of the patent by its use, a 4 per cent. cent. solution being the strength generally employed. Inject in and around the part ; allow five minutes before operating.—*College and Clinical Record.*

**Fifty** thousand tons of soot are taken from the chimneys of London annually. It is valued at two hundred thousand dollars, and is used for fertilizing purposes.

**Horses and Cows** can be protected from flies by sponging them lightly with soap-suds to which has heen added a trace of carbolic acid.

